# Longitudinal association between obesity and periodontal diseases among secondary school students in Hong Kong: a prospective cohort study

**DOI:** 10.1186/s12903-018-0659-3

**Published:** 2018-11-20

**Authors:** Ling-Wei Li, Hai Ming Wong, Colman P. McGrath

**Affiliations:** 1 Paediatric Dentistry & Orthodontics, Faculty of Dentistry, The University of Hong Kong, Prince Philip Dental Hospital, 34 Hospital Road, Hong Kong, SAR China; 2 Periodontology & Public health, Faculty of Dentistry, The University of Hong Kong, Prince Philip Dental Hospital, 34 Hospital Road, Hong Kong, SAR China

**Keywords:** Child dentistry, Oral health, Cohort studies, Adiposity

## Abstract

**Background:**

There is no consensus opinion regarding the association between obesity and periodontal diseases among children and adolescents in the literature.

**Methods:**

A prospective observational cohort study was conducted in a Hong Kong cohort at age 12, 15 and 18. CPI and various obesity indices including BMI, WC, WHR, WHtR, and TRSKF were recorded during each wave of data collection. Information on socioeconomic status and oral health behaviors were collected through self-completed questionnaires.

**Results:**

Two hundred and eighty-two (male: 122 and female: 160) participants completed all three rounds of data collection. Prevalence of overweight/obesity was 27.0, 19.1, and 14.2% at 12, 15, and 18 years, respectively. 19.9% participants had healthy periodontal conditions at age 12. While the percentage dropped to 10.3% at 15 years and 5.7% at 18 years. The proportion of 15-year-old adolescents who brushed teeth more than twice a day was significantly higher among participants belonging to the lower BMI, WC, WHR, and WHtR category (*P* < 0.05). The fully adjusted model revealed that participants with lower BMI at age 15 had higher probability of having more than 50% index teeth free from periodontal diseases at age 18 (OR: 2.78; 95% CI: 1.16, 6.64; *P* = 0.022).

**Conclusions:**

Although higher BMI at 15 years was associated with more extensive periodontal inflammation at age 18, this was believed to be an indirect association confounded by the poor oral health care among overweight/obese individuals. Oral health promotions should be directed to improve periodontal conditions of overweight/obese secondary school students.

## Background

Childhood obesity is an increasing concern not only in developed countries but also in low- and middle-income countries. In most regions of the world, more deaths were associated with overweight than with underweight [1]. The negative effect of childhood obesity on general health has been extensively studied [2]. Investigations into the association between obesity and periodontal diseases has also attracted researchers’ attention. Laboratory studies at molecular and cellular level have suggested bidirectional relationships between obesity and periodontal diseases [3]. Further evidence of the association comes from a meta-analysis [4] that demonstrated higher probability of periodontal diseases among obese individuals in adults (OR (Odds Ratio): 1.35; 95% CI (Confidence Interval): 1.23, 1.47). However, it is noteworthy that this review also highlighted the reliance on cross-sectional data in existing studies, which failed to provide temporal ordering of the events [4]. The association between obesity and periodontal diseases is further complicated by Zuza et al.’s finding that obesity did not interfere recovery of periodontal tissues or decrease of cytokines after non-surgical periodontal treatments in adults [5].

The association between obesity and periodontitis is more obscure among children and adolescents. Modéer et al. showed that obesity was associated with reduced flow rate of whole saliva and increased Visual Plaque Index (VPI) and Bleeding on Probing (BOP) among 14-year-old adolescents [6]. On the contrary, Petti et al. argued that there was no difference in prevalence of overweight between girls with and without gingivitis [7]. A systemic review found that existing studies were unable to provide consensus opinion regarding the association between adiposity and periodontal diseases among children and adolescents [8].

Body Mass Index (BMI) is the most frequently used index of obesity in epidemiological studies. However, the link between percent body fat and BMI is not strong [9]. Furthermore, it has been suggested that BMI is insensitive in identifying children with excessive adiposity [10]. Consequently, various indices were developed to provide more comprehensive assessment of accumulation and distribution of body fat. Waist Circumference (WC), Waist-Hip Ratio (WHR), and Waist-Height Ratio (WHtR) [11] are specific measures of central obesity and are related to risk of cardiovascular diseases [12]. Triceps Skinfold Thickness (TRSKF) is a widely employed indicator of peripheral obesity [13]. Most current studies take BMI as the only measurement of obesity. Peng et al. [14] suggested that more adiposity indices should be included in future studies.

Due to the lack of understanding of the exact extent and temporal ordering of the association between adiposity status and periodontal diseases among children and adolescents, there is a distinct urge for prospective cohort studies addressing this problem. In addition, it is desirable to assess various adiposity indices and adjust for the effect of potential confounders. Based on a sample of Chinese in Hong Kong, the aim of the present study was to assess changes of adiposity indices and periodontal status from 12 through 15 to 18 years and to examine their longitudinal associations.

## Methods

### Study design and study population

A random sample of secondary school students who were born between April 1st to May 31st, 1997 were selected in 2010 from local schools in Hong Kong. The follow-ups of this prospective, longitudinal cohort study were conducted in 2013 and 2015 when the participants reached the age of 15 and 18 years, respectively. The statistical power was set at 80% with response rate was set at 75%, according to the ORs which reported in the study of Modeer et al. [6] and oral health status in 2001 among Hong Kong 12-year-olds [15], 650 participants were needed for the baseline sample size. Students with severe systematic diseases and history of orthodontic treatment were excluded. Anthropometric measurements and periodontal assessments were performed; participants’ socioeconomic status and oral-health-related behaviors were also collected in each round.

### Ethics, consent and permissions

The ethical approval of this study was granted by the Institutional Review Board of the University of Hong Kong/Hospital Authority Hong Kong West Cluster (UW 15–178). A written consent from 18-year-old students, and parents/primary caregivers of 12- and 15-year-old students, and a verbal consent from 12- and 15-year-old students were obtained from all participants.

### Measurements

#### Anthropometric measurements (exposures and outcomes)

Body height, body weight, WC, Hip Circumference (HC), and TRSKF were measured with light clothing and no shoes by trained and calibrated examiners following standardized protocol suggested by Lohman et al. [13]. Body height, WC, HC was measured to the nearest 0.1 cm, TRSKF was assessed to nearest 0.01 cm and body weight was recorded to the nearest 0.1 kg. Repeated assessment was performed among 10% of the participants to determine intra- and inter-examiner reliability. Obesity indices used included BMI (The cut-offs for BMI was based on International Obesity Task Force (IOTF) criteria [16]), WC, WHR, WHtR and TRSKF.

#### Periodontal assessment (exposures and outcomes)

Community Periodontal Index (CPI) was used to evaluate participants’ periodontal health following the World Health Organization (WHO) 1997 guideline (WHO 1997). The dentition was divided into six sextants. One index tooth was selected from each sextant (16, 11, 26, 36, 31, and 46). The index teeth were examined with a mouth mirror mounted with LED light and a WHO CPI probe. The CPI scores were recorded as follows: CPI = 0, normal; CPI = 1, bleeding on probing and no pocket ≥3.5 mm; CPI = 2, calculus present and no pocket ≥3.5 mm; CPI = 3, a shallow pocket with depth of 3.5–5.5 mm; and CPI = 4, a deep pocket depth ≥ 5.5 mm. An approximately 20 g probing force was applied during periodontal examination. At age 12, only scores of 0, 1, and 2 were given. At age 15 and 18, all five scores were used. The highest CPI score among the six index teeth was used to indicate periodontal status of a participant. Examinations at three waves of data collection were conducted by the same two trained and calibrated dentists. Ten percent of randomly selected participants were re-examined for assessment of intra- and inter-examiner reliability.

#### Socioeconomic status and oral health-related behavior collection (exposures)

Participants’ socioeconomic status (parental employment status and family income) and oral health-related behaviors (frequency of tooth brushing) were collected by self-completed questionnaires for them and their caregivers. The question used in questionnaire to assess frequency of teeth brushing was “How often do you brush your teeth each day?” and the answers could be chosen from “less than once”, “once”, “twice” and “more than twice”.

### Statistical analysis

Differences in baseline distribution of gender, parental employment status, family income, frequency of tooth brushing, and CPI score were compared between responders and non-responders through Cohen’s w test. Distributional differences in BMI, WC, WHR, WHtR, and TRSKF were compared using Cohen’s d test. Continuous data were described using mean, standard deviation (SD), and median; categorical variables were described using frequency. Friedman’s two-way analysis of variance was used for comparison of anthropometric measurements among 12, 15, and 18 years. Temporal changes in distribution of CPI were explored through Cochran’s Q test.

For further investigation of the association between anthropometric variables and oral health status, BMI was used to dichotomize participants into underweight/normal weight group and overweight/obesity group. Besides, participants with ≤50% index teeth with CPI = 0 were considered to have more extensive periodontal inflammation while participants with > 50% index teeth with CPI = 0 were considered to be in the less extensive periodontal inflammation group. In addition, for each gender, participants were dichotomized into lower and upper 50th percentile according to their WC, WHR, WHtR, and TRSKF. For each wave of data collected, bivariate relationships of anthropometric variables and CPI with independent variables (gender, parental employment status, family income, and frequency of tooth brushing) were tested using Chi-square tests.

Binary logistic regression was performed to evaluate the longitudinal association between adiposity indices and CPI. All the variables were classified as mentioned before. Three models were employed in all binary logistic regressions. In Model 1, only gender was adjusted. In Model 2, gender and socioeconomic factors (parental employment status and family income) were adjusted. In Model 3, gender, socioeconomic factors and behavioral factors (frequency of tooth brushing) were adjusted. Intra- and inter-examiner reliability of anthropometric data were examined by intra-class correlation coefficient (ICC). Intra- and inter-examiner reliability of scoring of CPI were assessed using Kappa statistics. All statistical analyses were performed using SPSS 23.0 (IBM Corp., USA). The level of statistical significance was set at 0.05 for all analyses.

## Results

A total of 668 12-year-old participants (male: 329 and female: 339) were recruited from the baseline examination. Of them, 436 (male: 211 and female: 225) participated in the first follow-up at age 15 years and 383 (male: 168 and female: 215) participated in the second follow-up at age 18 years. There were 282 (male: 122 and female: 160) participants who completed all three stages of data collection. All analyses were performed based on data obtained from these 282 participants. The follow-up participants had similar sociodemographic, anthropometric and CPI characteristics as the lost participants (Cohen’s effect size < 0.3) (Table 6 in Appendix). There was a consistent trend of increase in mean and median BMI during the observation period, but the increase from 15 to 18 years was not statistically significant (Table 7 in Appendix). Among the 12-year-olds, 27.0% were overweight or obese. The prevalence figure dropped to 14.2% at 18 years (Table 8 in Appendix; Table 1). WC, WHR, and WHtR at 18 years were significantly lower than at pervious two age periods (Table 7 in Appendix). All obesity indices were significantly associated with frequency of tooth brushing at 15 years (*P* < 0.05) (Table 1). More specifically, participants who brushed their teeth less than twice a day had significant higher probability of being in the upper 50th percentile of WC (62.2%), WHR (62.2%), and WHtR (66.2%) than those who brushed teeth more frequently. More frequent tooth brushing was associated with lower probability of overweight/obesity at 15 years (*P* < 0.001) (Table 1).

**Table 1:** Association of adiposity and periodontal status with sociodemographic and oral health behavioral factors at 15 years

Variable	n (%)	BMI		WC		WHR		WHtR		TRSKF		CPI	
		Underweight+ normal weight n (%)	Overweight + Obese n (%)	≤median n (%)	>median n (%)	≤median n (%)	>median n (%)	≤median n (%)	>median n (%)	≤median n (%))	>median n (%)	≤ 50% index teeth with CPI = 0 n (%)	> 50% index teeth with CPI = 0 n (%)
Gender			*P* = 0.020*		*P* = 0.974		*P* = 1.000		*P* = 0.958		*P* = 0.603		*P* = 0.002**
Male	122 (43.3%)	91 (74.6%)	31 (25.4%)	62 (50.8%)	60 (49.2%)	61 (50.0%)	61 (50.0%)	61 (50.0%)	61 (50.0%)	61 (50.0%)	61 (50.0%)	82 (67.2%)	40 (32.8%)
Female	160 (56.7%)	137 (85.6%)	23 (14.4%)	81 (50.6%)	79 (49.4%)	80 (50.0%)	80 (50.0%)	80 (50.3%)	79 (49.7%)	85 (53.1%)	75 (46.9%)	78 (48.8%)	82 (51.3%)
Parental employment status			*P* = 0.022*		*P* = 0.009**		*P* = 0.580		*P* = 0.184		*P* = 0.079		*P* = 0.139
Both employed	167 (62.5%)	128 (76.6%)	39 (23.4%)	76 (45.5%)	91 (54.5%)	81 (48.5%)	86 (51.5%)	79 (47.6%)	87 (52.4%)	80 (47.9%)	87 (52.1%)	99 (59.3%)	68 (40.7%)
At least one unemployed	100 (37.5%)	88 (88.0%)	12 (12.0%)	62 (62.0%)	38 (38.0%)	52 (52.0%)	48 (48.0%)	56 (56.0%)	44 (44.0%)	59 (59.0%)	41 (41.0%)	50 (50.0%)	50 (50.0%)
Family income			*P* = 0.295		*P* = 0.946		*P* = 0.081		*P* = 0.790		*P* = 0.204		*P* = 0.387
Less than HK$ 10,000	43 (16.2%)	37 (86.0%)	6 (14.0%)	23 (53.5%)	20 (46.5%)	21 (48.8%)	22 (51.2%)	20 (46.5%)	23 (53.5%)	24 (55.8%)	19 (44.2%)	26 (60.5%)	17 (39.5%)
HK$10,001-HK$30,000	144 (54.1%)	112 (77.8%)	32 (22.2%)	73 (50.7%)	71 (49.3%)	65 (45.1%)	79 (54.9%)	75 (52.4%)	68 (47.6%)	67 (46.5%)	77 (53.5%)	83 (57.6%)	61 (42.4%)
More than HK$ 30,000	79 (29.7%)	67 (84.8%)	12 (15.2%)	41 (51.9%)	38 (48.1%)	48 (60.8%)	31 (39.2%)	40 (50.6%)	39 (49.4%)	46 (58.2%)	33 (41.8%)	39 (49.4%)	40 (50.6%)
Frequency of tooth brushing			*P* < 0.001***		*P* = 0.010*		*P* = 0.015*		*P* = 0.001**		*P* = 0.087		*P* = 0.020*
Less than twice a day	74 (26.2%)	49 (66.2%)	25 (33.8%)	28 (37.8%)	46 (62.2%)	28 (37.8%)	46 (62.2%)	25 (33.8%)	49 (66.2%)	32 (43.2%)	42 (56.8%)	50 (67.6%)	24 (32.4%)
At least twice a day	208 (73.8%)	179 (86.1%)	29 (13.9%)	115 (55.3%)	93 (44.7%)	113 (54.3%)	95 (45.7%)	116 (56.0%)	91 (44.0%)	114 (54.8%)	94 (45.2%)	108 (51.9%)	100 (48.1%)

*CPI* community periodontal index, *BMI* body mass index, *WC* waist circumference, *WHR* waist-hip ratio, *WHtR* waist-height ratio, *TRSKF* triceps skinfold thickness

*P < 0.05, **P < 0.01, ***P < 0.001

*P* values were calculated through Chi-square tests

**Fig. 1:**
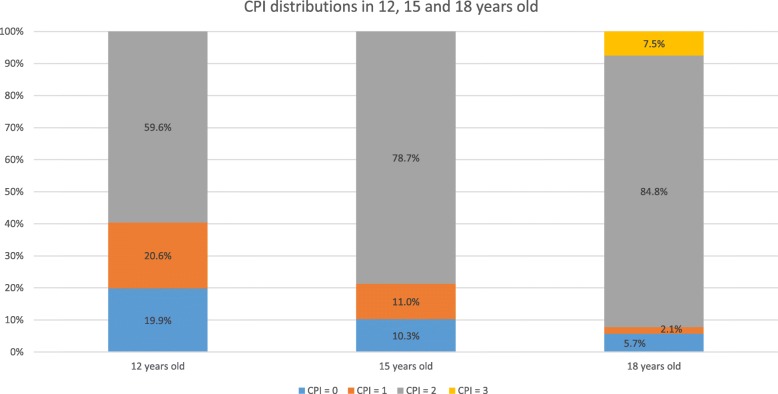
Distribution of participants according to levels of CPI at 12, 15, and 18 years

Distribution of CPI score at 12, 15, and 18 years was shown in Fig. 1. About 60% of participants had a score of 2 at 12 years. The prevalence increased by 19.2% at 15 years and by 25.2% at 18 years. In contrast, the prevalence of CPI = 0 decreased progressively from 19.9% at 12 years to 10.3% at 15 years and 5.7% at 18 years (Fig. 1). The proportion of participants with more extensive periodontal inflammation increased progressively from 42.2% through 56.0 to 67.7% during the observation period (*P* < 0.001) (Figure 1 in Appendix). The mean number of sextants with all index teeth free from periodontitis decreased from 3.1 to 2.4 from 12 to 18 years. During the study period, the mean number of sextants with index teeth having CPI of 2 increased by 1.1. Extent of periodontal inflammation was not associated with parental employment status, family income, and frequency of tooth brushing at 12 and 18 years (Tables 8 and 9 in Appendix). Among the 15-year-olds, participants who brushed teeth less than twice a day were more likely to suffer from more extensive periodontal inflammation than those who brushed teeth at least twice a day (*P* = 0.020) (Table 1). Table 1 also showed that more extensive periodontal inflammation was more prevalent (*P* = 0.002) among males (67.2%) than among females (48.8%). The ICC value for height, weight, WC, hip circumference, and TRSKF were between 0.94 and 1.00 (excellent). The kappa values of intra- and inter-examiner reliability for CPI scores ranged between 0.71 and 0.79 (good to excellent).

Longitudinal association between adiposity indices and periodontal status were investigated using binary logistic regression (Tables 2, 3, 4 and 5). The fully adjusted model (Model 3) in Table 2 revealed that participants in the lower BMI (underweight/normal weight) group had significantly higher probability (*P* = 0.022) of having more than 50% index teeth free from periodontal inflammation (OR: 2.78; 95% CI: 1.16, 6.64) than participants in the higher BMI group (overweight/obese group). Model 1 in Table 2 showed that the chance for participants with lower WHR (lower 50th percentile) to have less extensive periodontal inflammation was 1.75 times as likely (95% CI:1.05, 2.91) compared to those with higher WHR (upper 50th percentile). However, the association was no longer significant when possible confounders were included in Model 2 and Model 3 (Table 2). No longitudinal association was found between obesity indices at 12 years and CPI at 15 years (Table 6 in Appendix), CPI at 12 years and obesity indices at 15 years (Table 4), and CPI at 15 years and obesity indices at 18 years (Table 5).

**Table 2:** Longitudinal association between adiposity indices at 15 years and CPI at 18 years through binary logistic regression

Independent Variable	Model 1^a^			Model 2^b^			Model 3^c^		
	OR	95% CI	*P*	OR	95% CI	*P*	OR	95% CI	*P*
BMI	3.16	1.41, 7.04	0.005**	3.12	1.32, 7.39	0.009**	2.78	1.16, 6.64	0.022*
WC	1.47	0.89, 2.44	0.135	1.57	0.92, 2.69	0.099	1.47	0.85, 2.53	0.166
WHR	1.75	1.05, 2.91	0.031*	1.45	0.85, 2.47	0.170	1.34	0.78, 2.31	0.284
WHtR	1.33	0.80, 2.20	0.269	1.31	0.77, 2.22	0.322	1.17	0.68, 2.01	0.574
TRSKF	1.36	0.82, 2.26	0.228	1.32	0.78, 2.25	0.305	1.27	0.74, 2.18	0.380

*CPI* community periodontal index, *CI* conference interval; BMI, body mass index, *WC* waist circumference, *WHR* waist-hip ratio, *WHtR* waist-height ratio, *TRSKF* triceps skinfold thickness

Dependent variable: CPI at 18 years. Participants were classified by as having ≤50% index teeth with CPI = 0 vs >  50% index teeth with CPI = 0 (event group)

Independent variable: Adiposity indices at 15 years. BMI was used to classify participants into underweight/normal weight group vs overweight/obesity group (reference group). WC, WHR, WHtR, and TRSKF were used to classify participant according to their median values (participants in the upper 50th percentile constitute the reference group)

^a^Model 1: Adjusted for gender

^b^Model 2: Adjusted for socioeconomic factors (parental employment status and family income) at 15 years

^c^Model 3: Fully adjusted model. Model 2 plus behavioral factors (frequency of tooth brushing) at 15 years

*P < 0.05, **P < 0.01

**Table 3:** Longitudinal association between adiposity indices at 12 years and CPI at 15 years through binary logistic regression

Independent Variable	Model 1^a^			Model 2^b^			Model 3^c^		
	OR	95% CI	*P*	OR	95% CI	*P*	OR	95% CI	*P*
BMI	1.26	0.72, 2.20	0.422	1.13	0.64, 2.01	0.677	1.13	0.64, 2.01	0.678
WC	1.11	0.68, 1.79	0.682	0.96	0.58, 1.57	0.867	0.96	0.58, 1.57	0.855
WHR	0.94	0.58, 1.52	0.807	0.86	0.51, 1.37	0.475	0.83	0.51, 1.37	0.474
WHtR	1.00	0.62, 1.62	1.000	0.86	0.52, 1.42	0.557	0.86	0.52, 1.41	0.545
TRSKF	1.46	0.80, 2.67	0.222	1.37	0.73, 2.58	0.331	1.31	0.69, 2.49	0.406

*CPI* community periodontal index, *CI* conference interval, *BMI* body mass index, *WC* waist circumference, *WHR* waist-hip ratio, *WHtR* waist-height ratio, *TRSKF* triceps skinfold thickness

Dependent variable: CPI at 15 years. Participants were classified by as having ≤50% index teeth with CPI = 0 vs >  50% index teeth with CPI = 0 (event group)

Independent variable: Adiposity indices at 12 years. BMI was used to classify participants into underweight/normal weight group vs overweight/obesity group (reference group). WC, WHR, WHtR, and TRSKF were used to classify participant according to their median values (participants in the upper 50th percentile constitute the reference group)

^a^Model 1: Adjusted for gender

^b^Model 2: Adjusted for socioeconomic factors (parental employment status and family income) at 12 years

^c^Model 3: Fully adjusted model. Model 2 plus behavioral factors (frequency of tooth brushing) at 12 years

*P < 0.05, **P < 0.01

**Table 4:** Longitudinal association between CPI at 12 years and adiposity indices at 15 years through binary logistic regression

Dependent Variable	Model 1^a^			Model 2^b^			Model 3^c^		
	OR	95% CI	*P*	OR	95% CI	*P*	OR	95% CI	*P*
BMI	1.25	0.69, 2.29	0.462	1.27	0.68, 2.38	0.452	1.26	0.67, 2.35	0.477
WC	1.29	0.80, 2.07	0.295	1.34	0.81, 2.20	0.252	1.32	0.81, 2.18	0.269
WHR	1.55	0.96, 2.49	0.071	1.43	0.88, 2.34	0.152	1.41	0.86, 2.32	0.173
WHtR	1.28	0.80, 2.06	0.309	1.24	0.76, 2.02	0.396	1.22	0.74, 2.00	0.435
TRSKF	1.24	0.77, 1.99	0.378	1.19	0.73, 1.95	0.480	1.19	0.73, 1.94	0.496

*CPI* Community Periodontal Index, *CI* conference interval, *BMI* body mass index, *WC* waist circumference, *WHR* waist-hip ratio, *TRSKF* triceps skinfold thickness

Dependent variable: Adiposity indices at 15 years. Participants were classified into underweight/normal weight group vs overweight/obese group (event group) according to BMI. WC, WHR, WHtR and TRSKF dichotomized participants at median (participants in the upper 50th percentile constitute the reference group)

Independent variable: CPI at 12 years. Participants were classified by as having ≤50% index teeth with CPI = 0 vs >  50% index teeth with CPI = 0 (reference group)

^a^Model 1: Adjusted for gender

^b^Model 2: Adjusted for socioeconomic factors (parental employment status and family income) at 12 years

^c^Model 3: Fully adjusted model. Model 2 plus behavioral factors (frequency of tooth brushing) at 12 years

* *P* < 0.05, ** *P* < 0.01

**Table 5:** Longitudinal association between CPI at 15 years and adiposity indices at 18 years through binary logistic regression

Dependent Variable	Model 1^a^			Model 2^b^			Model 3^c^		
	OR	95% CI	*P*	OR	95% CI	*P*	OR	95% CI	*P*
BMI	1.15	0.57, 2.34	0.701	1.01	0.48, 2.13	0.973	0.95	0.45, 2.02	0.895
WC	1.39	0.86, 2.25	0.181	1.25	0.76, 2.07	0.380	1.19	0.72, 1.98	0.504
WHR	1.13	0.70, 1.82	0.625	1.13	0.69, 1.87	0.633	1.06	0.64, 1.77	0.814
WHtR	1.27	0.79, 2.06	0.328	1.22	0.74, 2.02	0.428	1.17	0.71, 1.94	0.543
TRSKF	1.43	0.88, 2.32	0.146	1.34	0.81, 2.22	0.258	1.32	0.80, 2.19	0.280

*CPI* community periodontal index, *CI* conference interval, *BMI* body mass index, *WC* waist circumference, *WHR* waist-hip ratio, *TRSKF* triceps skinfold thickness

Dependent variable: Adiposity indices at 18 years. Participants were classified into underweight/normal weight group vs overweight/obese group (event group) according to BMI. WC, WHR, WHtR and TRSKF dichotomized participants at median (participants in the upper 50th percentile constitute the reference group)

Independent variable: CPI at 15 years. Participants were classified by as having ≤50% index teeth with CPI = 0 vs >  50% index teeth with CPI = 0 (reference group)

^a^Model 1: Adjusted for gender

^b^Model 2: Adjusted for socioeconomic factors (parental employment status and family income) at 15 years

^c^Model 3: Fully adjusted model. Model 2 plus behavioral factors (frequency of tooth brushing) at 15 years

* *P* < 0.05, ** *P* < 0.01

## Discussion

In this study, WC, WHR, and WHtR were found to decrease with growth. At 18 years, the proportion of girls who were underweight was 26.3%, which was higher than the prevalence in the IOTF criteria. Underweight is prevalent among adolescents and emerging adults in Hong Kong. A previous study in Hong Kong reported that 35.9% girls and 27.5% boys aged 15–20 years were underweight [17]. The prevalence of underweight in Hong Kong may be associated with social preference towards thinner figures in local culture [18]. This study showed that most of the adiposity indices were not related to sociodemographic characteristics (Table 1, Tables 8 and 9 in Appendix). Similar findings were reported by Schooling et al. who found no associations between childhood obesity and family socioeconomic status in Hong Kong [19].

Substantial variations exist regarding CPI of adolescents among countries and among different surveys in one country. The proportion of adolescents free from periodontal inflammation ranged from 0 to 77% at 15 years and from 0 to 21% at 18 years [20]. The proportion observed in this study (10.3% at 12 years and 5.7% at 15 years) was well within the range reported previously. In the 2011 oral health survey in Hong Kong, 13.8% of 12-year-olds were free from periodontal inflammation [15], while the number was 19.8% in this study. We found that periodontal status of the participant got worse with growth. The proportion of participants free from periodontal diseases decreased from 19.9% at 12 years to 5.7% at 18 years. Furthermore, the mean number of sextants with CPI = 0 decreased from 3.1 to 2.4 during the study period. In spite of the decreasing trend, the number at 15 and 18 years (Table 10 in Appendix) were still higher than that reported in the survey by Hong Kong Government in the 1980s.

We found that 15-year-old participants who were underweight/of normal weight were less likely to have more extensive periodontal inflammation at 18 years (Table 2). However, we consider this to be an indirect association confounded by the negligence of oral health care among the overweight/obese participants. Analyses of data at 15 years demonstrated that the proportion of overweight/obesity was significantly higher among participants who brushed their teeth less than twice a day (33.8%) than among those who brushed their teeth more frequently (13.9%). The poor oral health behavior might account for the compromised periodontal condition at 18 years. Further evidence of the artefact of oral health behavior in the observed association came from the lack of association between BMI at 12 years and periodontal status at 15 years when there was no association between tooth brushing frequency and BMI at 12 years. Our belief was supported by Hujoel et al.’s proposition that the association between general and oral health was confounded by the effect of health behaviors [21, 22].

Laboratory studies have also been performed to investigate the association between obesity and periodontitis. Glycemic control and secretion of pro-inflammatory agents were recognized as the link among obesity, diabetes, and periodontitis [23]. Animal studies by Li et al. indicated that saturated fatty acid contributes to both Metabolic Syndrome and exacerbated alveolar bone loss [24]. However, it should be reminded that investigations into biological mechanisms of the association were mostly performed using samples of participants with obesity-related diseases such as diabetes and Metabolic Syndrome [25]. However, participants with systemic diseases were excluded from this study. This might explain the lack of association between obesity and periodontitis in this study in spite of the possible mechanisms established through laboratory studies.

Several cross-sectional studies [26] have suggested positive association between obesity and periodontitis. However, reliance on cross-sectional data provided limited information on temporal ordering of the association [27]. Furthermore, these studies mostly failed to account for the potential effect of confounders such as oral health habits. It should also be noticed that most studies explored the association between obesity and periodontitis among adults. In our sample of secondary school students, little clinical signs of chronic periodontitis could be seen. Therefore, specific clinical parameters of periodontitis such as attachment loss and pocket depth were not appropriate for use in the present study. This may be one of the possible reasons of the lack of associations between obesity and periodontitis in this study.

Clinical parameters used in existing studies were highly heterogeneous [8]. In this study, CPI was used as the indicator for periodontal status. Using CPI as a periodontal parameter may underestimate the diagnosis of periodontal diseases, because it is usually used to measure the periodontium around index teeth rather than to measure the periodontium around all teeth. In addition, CPI ignores measurement of the attachment loss; instead it measures the pocket depth. However, no pocket depth was identified among participants at age 12 and 15. Furthermore, only 5.7% of 18-year-old participants which was 16 adolescents were free from periodontal inflammation. Therefore, CPI = 0 and CPI = 3 were not employed as the cut-off points in this study. On the other hand, only six index teeth instead of every single tooth were examined in our surveys, which might comprised the accuracy of the results. In addition, due to the lack of internationally recognized cut-offs for WC, WHR, WHtR, and TRSKF among children and adolescents [28], we employed median as our cut-off point, which might differ from some other studies. The evident low proportion of obese subjects is another limitation in the present study.

## Conclusions

In this study, we found that overweight/obese secondary school students showed worse oral health awareness compared to underweight/normal weight individuals and higher BMI at age 15 was associated with more severe periodontal conditions at age 18. We recommend inclusion of more indicators of adolescents’ gingival status such as BOP, VPI, and Plaque Index (PI) in future studies. It is also important to adjust for more oral health behavioral factors, such as dental flossing and frequency of dental visit in future analyses. Given the negligence of oral health care among overweight/obese secondary school students, oral health promotion programs are necessary to improve their periodontal conditions.

## Figures and Tables

**Fig. 2:**
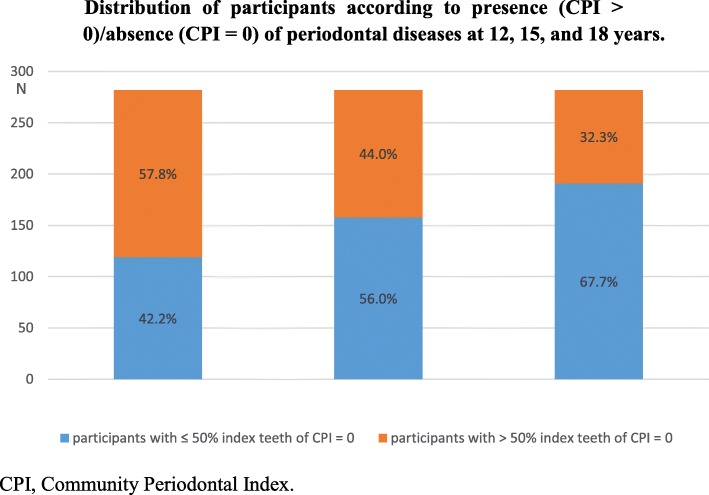
Distribution of participants according to presence (CPI > 0)/absence (CPI = 0) of periodontal diseases at 12, 15, and 18 years. CPI, Community Periodontal Index

## Appendix

**Table 6:** Comparison of baseline characteristics between follow-ups and those lost to follow-up

		Lost to follow-up Valid *n* (%)	Follow-ups Valid *n* (%)	Cohen’s effect size
Gender				0.10^a^
	Male	207 (53.6%)	122 (43.3%)	
	Female	179 (46.4%)	160 (56.7%)	
Parental employment status				0.03^a^
	Both employed	233 (62.5%)	180 (65.0%)	
	At least one unemployed	140 (37.5%)	97 (35.0%)	
Family income				0.06^a^
	Less than HK$ 10,000	94 (25.3%)	72 (26.5%)	
	HK$10,001-HK$30,000	175 (47.0%)	139 (51.1%)	
	More than HK$ 30,000	103 (27.7%)	61 (22.4%)	
Frequency of tooth brushing				0.06^a^
	Less than twice a day	119 (30.8%)	71 (25.2%)	
	At least twice a day	267 (69.2%)	211 (74.8%)	
Use of fluoride toothpaste				0.08^a^
	Yes	116 (30.1%)	107 (37.9%)	
	No or not sure	270 (69.9%)	175 (62.1%)	
Frequency of taking snack				0.02^a^
	Less than once a day	83 (21.5%)	62 (22.0%)	
	Once a day	195 (50.5%)	145 (51.4%)	
	At least twice a day	108 (28.0%)	75 (26.6%)	
CPI				0.06 ^a^
	≤ 50% index teeth with CPI = 0	188 (61.2%)	119 (38.8%)	
	> 50% index teeth with CPI = 0	198 (54.8%)	163 (45.2%)	
BMI	Valid n	386	282	0.09^b^
	Mean (SD)	19.99 (3.99)	19.66 (3.35)	
WC	Valid n	386	282	0.12^b^
	Mean (SD)	70.91 (9.90)	69.82 (8.62)	
WHR	Valid n	386	282	0.12^b^
	Mean (SD)	0.82 (0.06)	0.81 (0.05)	
WHtR	Valid n	386	282	0.13^b^
	Mean (SD)	0.45 (0.06)	0.45 (0.05)	
TRSKF	Valid n	208	179	0.04^b^
	Mean (SD)	10.54 (3.95)	10.69 (4.20)	
DMFT	Valid n	386	282	0.12^b^
	Mean (SD)	0.58 (1.04)	0.46 (0.94)	

*CPI* community periodontal index, *BMI* body mass index, *WC* waist circumference, *WHR* waist-hip ratio, *WHtR* waist-height ratio, *TRSKF* triceps skinfold thickness, *DMFT* the number of decayed, missing and filled permanent teeth

^a^Effects size based on Cohen’s w: 0.1 for small; 0.3 for medium; 0.5 for large effect

^b^Effects size based on Cohen’s h: 0.1 for small; 0.3 for medium; 0.5 for large effect

**Table 7:** Description of anthropometric measurements and DMFT at 12, 15, and 18 years

Variable	12-years-old (1)	15-years-old (2)	18-years-old (3)	*P*-value	Multiple comparison
	Mean (SD) Median	Mean (SD) Median	Mean (SD) Median		
BMI^a^				< 0.001***	(1) < (2) = (3)
*n* = 281	19.66 (3.36) 18.82 14.31–30.05	21.03 (3.43) 20.29 15.24–34.02	21.15 (3.60) 20.37 15.00–35.89		
WC^a^				< 0.001***	(3) < (1) < (2)
*n* = 282	69.82 (8.62) 67.65 52.50–100.10	72.53 (8.97) 70.20 57.4–104.50	68.86 (8.99) 67.00 53.0–111.00		
WHR^a^				< 0.001***	(3) < (2) < (1)
*n* = 282	0.81 (0.05) 0.81 0.67–0.97	0.79 (0.06) 0.78 0.63–0.98	0.77 (0.06) 0.76 0.58–1.09		
WHtR^a^				< 0.001***	(3) < (2) = (1)
*n* = 281	0.45 (0.05) 0.44 0.34–0.61	0.44 (0.05) 0.43 0.34–0.64	0.42 (0.05) 0.40 0.32–0.66		
TRSKF^a^				< 0.001***	(1) < (2) = (3)
*n* = 179	10.69 (4.20) 9.50 4.20–29.3	14.92 (6.66) 13.80 4.40–44.1	15.78 (6.29) 15.30 4.70–39.60		
	n (%) (≤ 50% index teeth with CPI = 0)	*n* (%) (≤ 50% index teeth with CPI = 0)	n (%) (≤ 50% index teeth with CPI = 0)		
CPI^b^				< 0.001***	(1) < (2) < (3)
*n* = 282	119 (42.2%)	158 (56.0%)	191 (67.7%)		

*BMI* body mass index, *WC* waist circumference, *HC* hip circumference, *WHR* waist-hip ratio, *WHtR* waist-height ratio, *TRSKF* triceps skinfold thickness, *DMFT* the number of decayed, missing and filled permanent teeth, *CPI* community periodontal index

^a^Non-parametric tests, related-samples Friedman’s two-way analysis of variance by ranks

^b^Non-parametric test, related-samples Cochran’s Q test

Valid number of cases of height in 15 years old was 281; valid number of cases of TRSKF in 12 years old was 179

**P* < 0.05, ***P* < 0.01, ****P* < 0.001

**Table 8:** Association of adiposity and periodontal status with sociodemographic and oral health behavioral factors at 12 years

Variable	n (%)	BMI		WC		WHR		WHtR		TRSKF		CPI	
		Underweight + normal weight n (%)	Overweight + Obese n (%)	≤median *n* (%)	>median n (%)	≤median *n* (%)	>median n (%)	≤median *n* (%)	>median *n* (%)	≤median n (%)	>median n (%)	≤ 50% index teeth with CPI = 0 n (%)	> 50% index teeth with CPI = 0 n (%)
Gender			*P* < 0.001***		*P* = 0.892		*P* = 1.000		*P* = 1.000		*P* = 0.953		*P* = 0.718
Male	122 (43.3%)	76 (62.3%)	46 (37.7%)	62 (50.8%)	60 (49.2%)	61 (50.0%)	61 (50.0%)	61 (50.0%)	61 (50.0%)	40 (53.3%)	35 (46.7%)	50 (41.0%)	72 (59.0%)
Female	160 (56.7%)	130 (81.3%)	30 (18.8%)	80 (50.0%)	80 (50.0%)	80 (50.0%)	80 (50.0%)	80 (50.0%)	80 (50.0%)	55 (52.9%)	49 (47.1%)	69 (43.1%)	91 (56.9%)
Parental employment status			*P* = 0.136		*P* = 0.454		*P* = 0.558		*P* = 0.935		*P* = 0.210		*P* = 0.679
Both employed	180 (65.0%)	126 (70.0%)	54 (30.0%)	88 (48.9%)	92 (51.1%)	88 (48.9%)	92 (51.1%)	90 (50.0%)	90 (50.0%)	59 (49.6%)	60 (50.4%)	77 (42.8%)	103 (57.2%)
At least one unemployed	97 (35.0%)	76 (78.4%)	21 (21.6%)	52 (53.6%)	45 (46.4%)	51 (52.6%)	46 (47.4%)	49 (50.5%)	48 (49.5%)	34 (59.6%)	23 (40.4%)	39 (40.2%)	58 (59.8%)
Family income			*P* = 0.418		*P* = 0.461		*P* = 0.523		*P* = 0.278		*P* = 0.335		*P* = 0.061
Less than HK$ 10,000	72 (26.5%)	56 (77.8%)	16 (22.2%)	35 (48.6%)	37 (51.4%)	33 (45.8%)	39 (54.2%)	34 (47.2%)	38 (52.8%)	24 (61.5%)	15 (38.5%)	38 (52.8%)	34 (47.2%)
HK$10,001-HK$30,000	139 (51.1%)	97 (69.8%)	42 (30.2%)	67 (48.2%)	72 (51.8%)	70 (50.4%)	69 (49.6%)	66 (47.5%)	73 (52.5%)	50 (52.6%)	45 (47.4%)	57 (41.0%)	82 (59.0%)
More than HK$ 30,000	61 (22.4%)	46 (75.4%)	15 (24.6%)	35 (57.4%)	26 (42.6%)	34 (55.7%)	27 (44.3%)	36 (59.0%)	25 (41.0%)	17 (44.7%)	21 (55.3%)	20 (32.8%)	41 (67.2%)
Frequency of tooth brushing			*P* = 0.789		*P* = 0.450		*P* = 0.891		*P* = 0.337		*P* = 0.413		*P* = 0.399
Less than twice a day	71 (25.2%)	51 (71.8%)	20 (28.2%)	33 (46.5%)	38 (53.5%)	35 (49.3%)	36 (50.7%)	32 (45.1%)	39 (54.9%)	21 (47.7%)	23 (52.3%)	33 (46.5%)	38 (53.5%)
At least twice a day	211 (74.8%)	155 (73.5%)	56 (26.5%)	109 (51.7%)	102 (48.3%)	106 (50.2%)	105 (49.8%)	109 (51.7%)	102 (48.3%)	74 (54.8%)	61 (45.2%)	86 (4.08%)	125 (59.2%)

*CPI* community periodontal index, *BMI* body mass index, *WC* waist circumference, *WHR* waist-hip ratio, *WHtR* waist-height ratio, *TRSKF* triceps skinfold thickness

**P* < 0.05, ***P* < 0.01, ****P* < 0.001. *P* values were calculated through Chi-square tests

**Table 9:** Association of adiposity and periodontal status with sociodemographic and oral health behavioral factors at 18 years

Variable	n (%)	BMI		WC		WHR		WHtR		TRSKF		CPI	
		Underweight + normal weight n (%)	Overweight + Obese n (%)	≤median *n* (%)	>median *n* (%)	≤median n (%)	>median n (%)	≤median n (%)	>median n (%)	≤median n (%)	>median n (%)	≤ 50% index teeth with CPI = 0 n (%)	> 50% index teeth with CPI = 0 n (%)
Gender			*P* = 0.008**		*P* = 0.917		*P* = 1.000		*P* = 1.000		*P* = 0.835		*P* = 0.167
Male	122 (43.3%)	97 (79.5%)	25 (20.5%)	61 (50.0%)	61 (50.0%)	61 (50.0%)	61 (50.0%)	61 (50.0%)	61 (50.0%)	61 (50.0%)	61 (50.0%)	88 (72.1%)	34 (27.9%)
Female	160 (56.7%)	145 (90.6%)	15 (9.4%)	81 (50.6%)	79 (49.4%)	80 (50.0%)	80 (50.0%)	80 (50.0%)	80 (50.0%)	82 (51.3%)	78 (48.8%)	103 (64.4%)	57 (35.6%)
Parental employment status			*P* = 0.042*		*P* = 0.848		*P* = 0.592		*P* = 0.443		*P* = 0.123		*P* = 0.087
Both employed	117 (61.3%)	95 (81.2%)	22 (18.8%)	60 (51.3%)	57 (48.7%)	60 (51.3%)	57 (48.7%)	62 (53.0%)	55 (47.0%)	53 (45.3%)	64 (54.7%)	85 (72.6%)	32 (27.4%)
At least one unemployed	74 (38.7%)	68 (91.9%)	6 (8.1%)	39 (52.7%)	35 (47.3%)	35 (47.3%)	39 (52.7%)	35 (47.3%)	39 (52.7%)	42 (56.8%)	32 (43.2%)	45 (60.8%)	29 (39.2)
Family income			*P* = 0.774		*P* = 0.910		*P* = 0.911		*P* = 0.931		*P* = 0.447		*P* = 0.056
Less than HK$ 10,000	23 (12.3%)	20 (87.0%)	3 (13.0%)	13 (56.5%)	10 (43.5%)	11 (47.8%)	12 (52.2%)	12 (52.2%)	11 (47.8%)	12 (52.2%)	11 (47.8%)	16 (69.6%)	7 (30.4%)
HK$10,001-HK$30,000	99 (53.0%)	83 (83.8%)	16 (16.2%)	51 (51.5%)	48 (48.5%)	49 (49.5%)	50 (50.5%)	49 (49.5%)	50 (50.5%)	45 (45.5%)	54 (54.5%)	74 (74.7%)	25 (25.3%)
More than HK$ 30,000	65 (34.8%)	57 (87.7%)	8 (12.3%)	34 (52.3%)	31 (47.7%)	34 (52.3%)	31 (47.7%)	34 (52.3%)	31 (47.7%)	36 (55.4%)	29 (44.6%)	37 (56.9%)	28 (43.1%)
Frequency of tooth brushing			*P* = 0.256		*P* = 0.062		*P* = 0.109		*P* = 0.180		*P* = 0.947		*P* = 0.147
Less than twice a day	84 (29.9%)	69 (82.1%)	15 (17.9%)	35 (41.7%)	49 (58.3%)	36 (42.9%)	48 (57.1%)	37 (44.0%)	47 (56.0%)	43 (51.2%)	41 (48.8%)	62 (73.8%)	22 (26.2%)
At least twice a day	197 (70.1%)	172 (87.3%)	25 (12.7%)	106 (53.8%)	91 (46.2%)	105 (53.3%)	92 (46.7%)	104 (52.8%)	93 (47.2%)	100 (50.8%)	97 (49.2%)	128 (65.0%)	69 (35.0%)

*CPI* community periodontal index, *BMI* body mass index, *WC* waist circumference, *WHR* waist-hip ratio, *WHtR* waist-height ratio, *TRSKF* triceps skinfold thickness

**P* < 0.05, ***P* < 0.01, ****P* < 0.001. *P* values were calculated through Chi-square tests

**Table 10:** Distribution of CPI scores by age and comparison with previous surveys

Age	*N*	Mean number of sextants with			
		CPI = 0	CPI = 1	CPI = 2	CPI = 3
12	282	3.1	0.7	2.2	–
15	282	3.0	0.7	2.3	0.0
18	282	2.4	0.2	3.3	0.1
Oral health surveys of Hong Kong Government					
12 [27] (2011)	1054	3.5	2.1	0.4	–
15–19 [38] (1982)	374	2.3	3.7	2.7	0.3
15–19 [39] (1984)	563	1.1	4.9	4.4	0.5

## Abbreviations

BMI: Body mass index
BOP: Bleeding on probing
CI: Confidence interval
CPI: Community periodontal index
HC: Hip circumference
IOTF: International Obesity Task Force
OR: Odds ratio
PI: Plaque index
SD: Standard deviation
TRSKF: Triceps skinfold thickness
VPI: Visual plaque index
WC: Waist circumference
WHO: World Health Organization
WHR: Waist-hip ratio
WHtR: Waist-height ratio
